# Increase in fall-related hospitalization, mortality, and lethality among older adults in Brazil

**DOI:** 10.11606/s1518-8787.2020054001691

**Published:** 2020-08-10

**Authors:** Lígia Raquel Ortiz Gomes Stolt, Daniel Vieira Kolisch, Clarice Tanaka, Maria Regina Alves Cardoso, Ana Carolina Basso Schmitt

**Affiliations:** I Universidade Federal da Paraíba Centro de Ciências da Saúde Departamento de Fisioterapia João PessoaPB Brasil Universidade Federal da Paraíba . Centro de Ciências da Saúde . Departamento de Fisioterapia . João Pessoa , PB , Brasil; II Universidade de São Paulo Faculdade de Medicina Departamento de Fisioterapia, Fonoaudiologia e Terapia Ocupacional São PauloSP Brasil Universidade de São Paulo . Faculdade de Medicina . Departamento de Fisioterapia, Fonoaudiologia e Terapia Ocupacional . São Paulo , SP , Brasil; III Universidade de São Paulo Faculdade de Saúde Pública Departamento de Epidemiologia São PauloSP Brasil Universidade de São Paulo . Faculdade de Saúde Pública . Departamento de Epidemiologia . São Paulo , SP , Brasil

**Keywords:** Older adults, Accidental Falls, Hospitalization, Mortality, Trends, Time series studies

## Abstract

**OBJECTIVE:**

To estimate the trends of fall-related hospitalization, mortality, and lethality among older adults in Brazil and regions.

**METHODS:**

This is a descriptive study based on data from the Hospital Information System of the Brazilian Unified Health System. We included records of every older adult, aged 60 years or older, hospitalized for accidental fall from January, 1998 to November, 2015 in all Brazilian regions. We selected the codes E885, E886, E880, E884, E884 from the International Classification of Diseases, 9th revision, and W01, W03, W10, W17, W18 from the 10th revision, and calculated fall-related hospitalization and mortality rates per 100,000 inhabitants, as well as lethality. To estimate trends, we applied the Prais-Winsten regression for time series analysis.

**RESULTS:**

During the period, 1,192,829 fall-related hospitalizations occurred, among which 54,673 had a fatal outcome; lethality was 4.5%. Hospitalization rates showed upward trends, with seasonality, in Brazil (11%), and in the Northeast (44%), Midwest (13%), and South regions (14%). The North showed a decreasing hospitalization rate (48%), and the Southeast a stationary one (3%).

**CONCLUSIONS:**

In Brazil, fall-related hospitalizations, mortality, and lethality among older adults showed an upward trend from 1998 to 2015, with seasonal peaks in the second and third quarters. Considering we are in plain demographic transition, to improve hospital healthcare and encourage falls prevention programs among older adults is essential.

## INTRODUCTION

Accidental Falls are unexpected events in which people come to rest on the ground, floor, or lower levels ^1^ . They affect 30% of older adults over 60 years, and 40 to 50% of seniors aged from 80 to 85 years. Among these groups (especially the long-lived), falls are the leading causes of injuries, fatal or not ^3^ . They are the most common type of unintentional injuries in the United States ^6^ , and the leading cause of accidental death. In Brazil, it is the third most common cause of accidental death ^3 , 7^ , and its prevalence ranged from 28.1% in 2011 to 25.1% to date ^8 , 9^ .

Accidental falls are more common at an old age and their sequelae may decrease functional independence and increase the risk of early death ^4^ ; thus, old age associated with natural depletion of physiological systems and functionality, increased morbidity, and early institutionalization turns accidental falls into a public health matter ^4^ . Besides its high cost, fall-related hospitalizations imply survival rates: only about 50% of older adults who fell and were admitted to hospitals will still be alive within one year ^10^ .

Victims of severe and moderate falls need medical or hospital assistance for treating sequelae ^11^ , leading to many hospital admissions that may or not require prolonged rehabilitation. In 2011, they accounted for 65% of emergency department visits owing to unintentional injuries in the United States ^6^ . In Australia, falls were responsible for 3% of older adults hospitalizations ^12^ ; in Brazil, they totaled 41% of hospitalizations due to external causes ^13^ .

Older adults aged 60 years or older are growing in number worldwide at an accelerated rate of 3% per year, increasing the risk of falls, and, consequently, sequelae, and fatalities ^14^ . In 2017, the world population was estimated at 7.6 billion people; 13% of them were older adults. It is projected that, by 2050, it will be composed of 9.8 billion people, 21% of which will be older adults. Simultaneously, Brazil will be among the 31 most populous countries in the world that, altogether, will concentrate 75% of the global population ^14^ .

As sequelae of moderate and severe falls often impair older adults’ independence and autonomy, the control and care with their occurrence is a major concern ^11^ . Knowing the distribution and handling of existing preventive measures for falls is important to optimize the planning of healthcare network, organize healthcare, and maintain adequate structure and human resources in the services. Considering that, this study aimed to estimate the trend and seasonality of fall-related hospitalization, mortality, and lethality rates among older adults in Brazil and regions.

## METHODS

For this time series study, we collected data from the HIS/SUS (Hospital Information System of the Brazilian Unified Health System) available in the Department of Informatics of the Brazilian Unified Health System (DATASUS) – a public database managed by the Ministry of Health, whose data use does not require ethical approval. We selected all hospital records of older adults aged 60 years or older, with paid authorization for inpatient hospital services (IHS), on hospitalizations and deaths occurred from January, 1998 to November, 2015. Records should present one of the following codes for accidental falls of the International Classification of Diseases (ICD), whether the 9th or 10th revision, as clinical diagnosis: E885, E886, E880, E884, E888 (ICD9), and W01, W03, W10, W17, W18 (ICD10). We included victims of severe falls requiring emergency care (treatment of fractures, head, or internal injuries), and moderate falls, requiring medical assistance (wounds, bruises, sprains, cuts, and abrasions) ^11^ .

We estimated hospitalization and mortality rates by age group and region per 100,000 inhabitants, as well as fall-related lethality rates. Lethality is understood as the proportion of death among the cases of a certain disease, indicating its severity within the population ^15^ . We also estimate the hospitalization rate among older adults for all causes in Brazil and regions, per 100,000 inhabitants, to estimate older adults’ tendency to access hospitals of the Brazilian Unified Health System (SUS). The number of hospitalizations (measured by IHS) for severe and moderate accidental falls in the period, as well as the resulting number of deaths, were collected in DATASUS’ HIS/SUS, by following the steps: Health information (TabNet) > Epidemiology and Morbidity > (HIS/SUS) Hospital Morbidity > “External causes, by place of residence,” considering the selected ICD codes. The population estimate of older adults (60 years and older) was obtained from the Brazilian Institute of Geography and Statistics, available at HIS/SUS: “Resident population” in Brazil and regions, both by age stratification according to decades (60–69, 70–79, 80 and older, or long-lived), and the overall group.

We estimated trends using the Prais-Winsten procedure for generalized linear regression, and evaluated the autocorrelation using the Durbin-Watson test. We also estimated the average growth rates, with 95% confidence intervals (95%CI) ^16^ . Data were imported to Microsoft Excel and tabulated and processed by v.13.0 Stata.

## RESULTS

In Brazil, the average fall-related hospitalization rate from 1998 to 2015 was 15.04/100,000 inhabitants/month; the average mortality rate for the same period was 0.67/100,000 inhabitants/month. The number of hospitalization of older adults in SUS for all causes was stationary in Brazil (-0.04%; 95%CI -0.11–0.02) and in its regions: North (-0,01%; 95%CI: -0,07–0,04), Northeast (-0,05%; 95%CI: -0,13–0,02), Southeast (-0,03%; 95%CI: -0,11–0,04), South (-0,03%; 95%CI: -0,09–0,01) and Midwest (-0,02%; 95%CI: -0,06–0,01).

**Figure 1:**
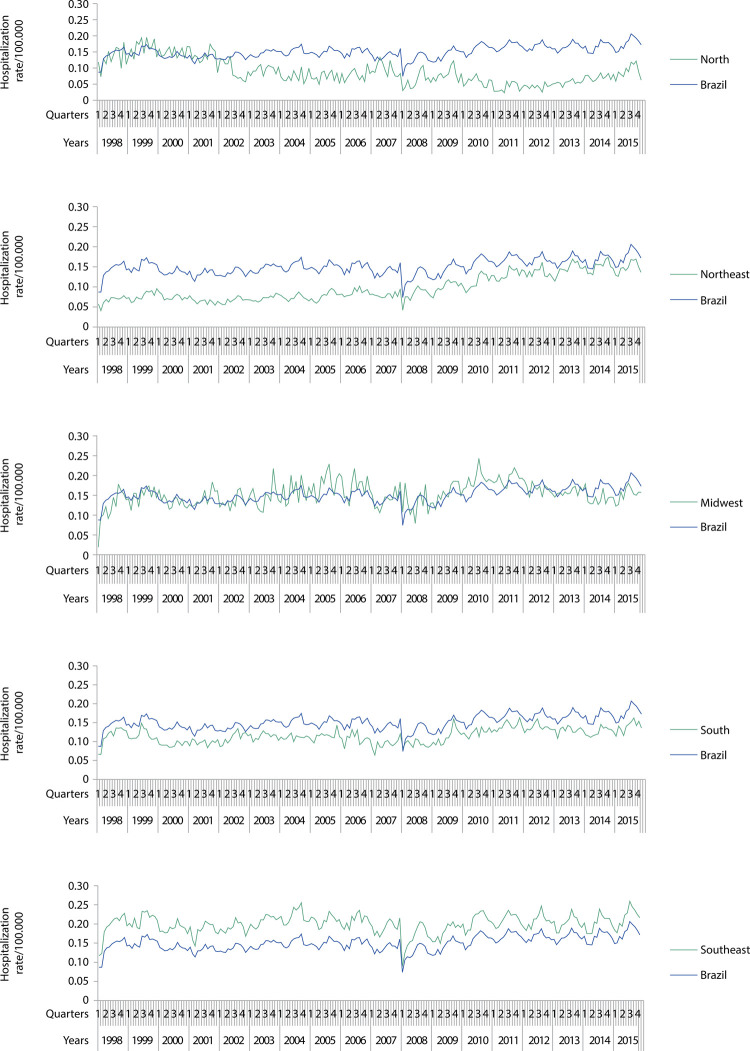
Hospitalization rate due to accidental falls among older adults in Brazil and regions. 1998–2015.

**Figure 2:**
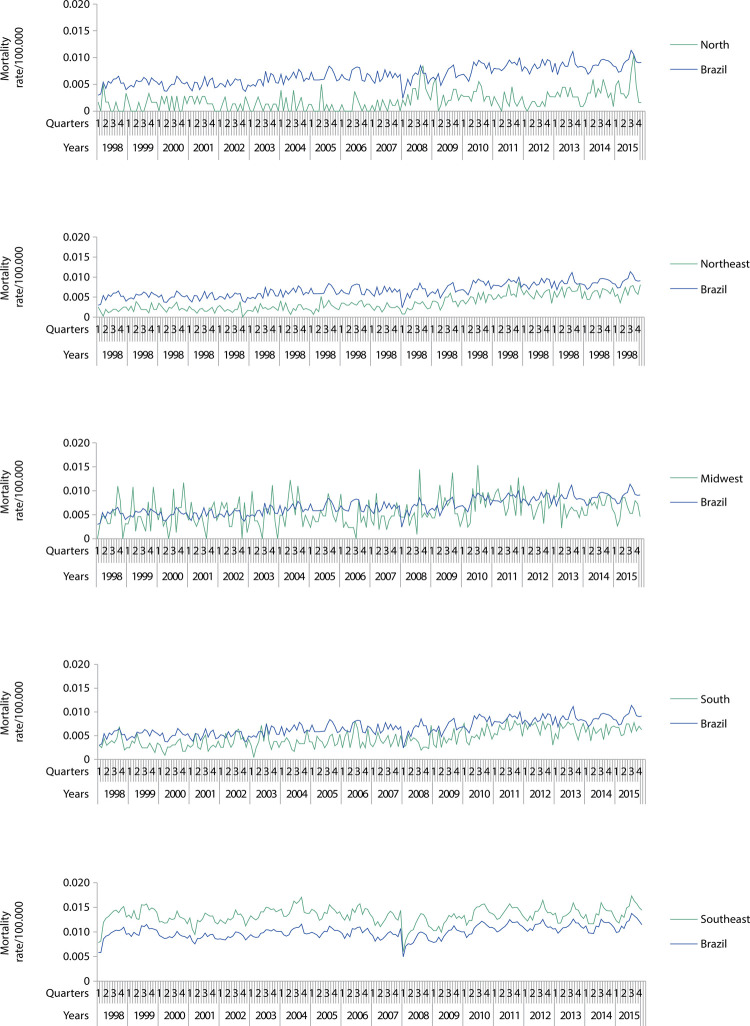
Mortality rate due to accidental falls among older adults in Brazil and regions. 1998–2015.

**Figure 3:**
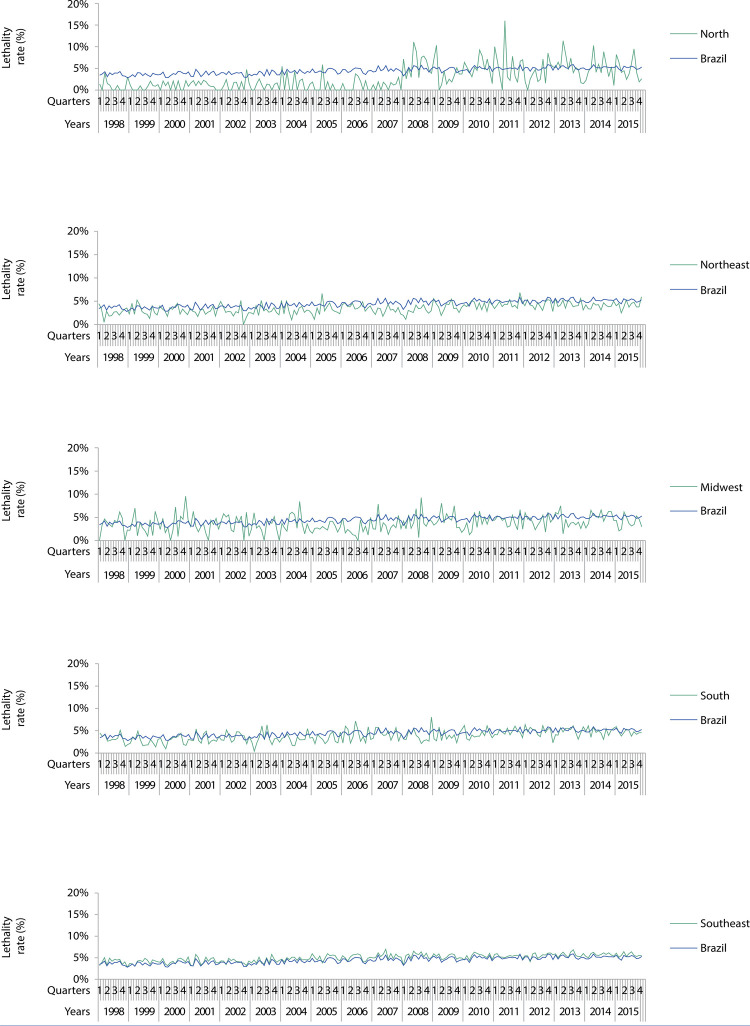
Lethality rate due to accidental falls among older adults in Brazil and regions. 1998–2015.

Figures 1, 2, and 3 show the magnitude of the rates, as well as their trend and seasonal variation. The Table shows the average growth rates for the studied indicators, enabling the identification of the type of trend (upward, downward, or stationary). Seasonality within age groups is indicated by the letter a.

**Table:** Annual growth rate of mortality, lethality, and hospitalization of older adults due to falls in Brazil and regions. 1998–2015.

		Annual growth rate (95%CI)		
		Hospitalization	Mortality	Lethality
Brazil	Total ^b^ /60 or +	0.11 (0.07 – 0.15) ^a^	0.32 (0.28 – 0.36) ^a^	0.22 (0.19– 0.24) ^a^
	60–69	0.12 (0.09 – 0.15) ^a^	0.17 (0.11 – 0.22) ^a^	0.05 (-0.00 – 0.10)
	70–79	0.11 (0.07 – 0.16) ^a^	0.27 (0.22 – 0.32) ^a^	0.16 (0.13 – 0.19)
	Long-lived ^c^	0.08 (0.01 – 0.16) ^a^	0.35 (0.30 – 0.40) ^a^	0.27 (0.24 – 0.31)
North	Total ^b^ /60 or +	-0.48 (-0,63 – -0.33)	0.19 (0.03 – 0.36)	0.68 (0.52 – 0.85)
	60–69	0.00 (-0.14 – 0.14)	0.32 (-0.44 – -0.20)	0.34 (-0.53 – -0.15)
	70–79	0.02 (-0.15 – -0.19)	0.12 (-0.25 – 0.02) ^a^	0.34 (-0.49 – -0.20)
	Long-lived ^c^	-1.00 (-1.27 – -0.72)	0.09 (-0.09 – 0.28)	1.03 (0.80 – 1.27)
Northeast	Total ^b^ /60 or +	0.44 (0.36 – 0.51) ^a^	0.71 (0.61 – 0.81) ^a^	0.27 (0.20 – 0.34)
	60–69	0.36 (0.29 – 0.43) ^a^	0.31 (0.16 – 0.47)	0.05 (-0,18 – 0.08)
	70–79	0.43 (0.35 – 0.50) ^a^	0.60 (0.47 – 0.73)	0.16 (0.07 – 0.26)
	Long-lived ^c^	0.55 (0.48 – 0.62) ^a^	0.93 (0.80 – 1.06) ^a^	0.04 (-0.07 – 0.14)
Midwest	Total ^b^ /60 or +	0.13 (0.06 – 0.19) ^a^	0.22 (0.12 – 0.33) ^a^	0.14 (0.04 – 0.24)
	60 to 69	0.15 (0.08 – 0.21) ^a^	0.19 (-0.32 – -0.06)	0.28 (-0.40 – -0.16)
	70 to 79	0.12 (0.05 – 0.19) ^a^	0.12 (-0.26 – 0.01)	0.18 (-0.31 – -0.06) ^a^
	Long-lived ^c^	0.62 (0.53 – 0.70) ^a^	0.12 (-0.03 – 0.27)	0.45 (-0.58 – -0.32)
Southeast	Total ^b^ /60 or +	0.03 (-0.01– 0.08) ^a^	0.23 (0.18 – 0.27) ^a^	0.20 (0.17 – 0.23) ^a^
	60–69	0.05 (-0.02 – 0.09) ^a^	0.13 (0.06 – 0.20) ^a^	0.08 (0.02 – 0.14) ^a^
	70–79	0.01 (-0.03 – 0.06) ^a^	0.19 (0.12 – 0.25) ^a^	0.18 (0.14 – 0.23)
	Long-lived ^c^	-0.07 (-0.14 – 0.00) ^a^	0.17 (0.11 – 0.22) ^a^	0.25 (0.21 – 0.29) ^a^
South	Total ^b^ /60 or +	0.14 (0.07 – 0.21) ^a^	0.44 (0.35 – 0.53)	0,31 (0.23 – 0.39)
	60–69	0.14 (0.08 – 0.19)	0.12 (-0.00 – 0.24)	0.00 (-0.12 – 0.11)
	70–79	1.25 (1.18 – 1.32) ^a^	0.31 (0.19 – 0.42)	0.18 (0.07 – 0.28)
	Long-lived ^c^	0.06 (-0.03 – 0.16) ^a^	0.45 (0.33 – 0.57)	0.40 (0.31 – 0.49)
Trend:		Stationary	Upward	Downward

^a^ seasonality / 95%CI: 95% confidence interval.

^b^ Total: total of older adults in the study, people aged 60 years or older.

^c^ Long-lived: older adults aged 80 years or older

In the study period, the national hospitalization, mortality, and lethality rates increased in most regions, except for lethality from 60 to 69 years, which decreased 5%. The second and third semesters presented seasonality, with peaks in hospitalization and mortality rates.

There were 1,192,829 fall-related hospitalizations in Brazilian public/affiliated hospitals. National hospitalization rate showed an upward trend for all age groups. Hospitalization rates were higher in the Southeast region than in the national region. Yet, we found most growth rates to indicate a stationary trend. The Midwest presented a hospitalization rate close to the national rate and an upward trend ( Figure 1 and Table ).

Hospitalization rates were lower in the North, Northeast, and South than in Brazil. In the North, it was stationary from 60 to 69 and from 70 to 79 years and showed a downward trend for the overall number of older adults and long-lived. The Northeast, as the South, showed an upward trend, stationary only for the long-lived group. Brazil and four of its five regions presented seasonal hospitalization rates for most or all age groups ( Figure 1 and Table ).

During the study period, 54,673 deaths due to accidental falls were reported. The overall mortality rate increased in all the regions and in Brazil, with an upward trend for every age group at national level. The Southeast was the only region with mortality rates higher than national rates; it also presented an upward trend for all age groups ( Figure 2 and Table ).

Mortality rates in the Midwest and South regions approached national rates. The Midwest showed an upward trend for the overall older population, and a downward for the 60-69 years age group. In the South, all age groups showed upward trends.

The mortality rates in the North and Northeast regions were lower than national rates. The North showed an upward trend for the overall older population, downward for the 60-69 years age group, and stationary up from 70 years. The Northeast showed an upward trend for all age groups, with seasonality in the long-lived group. ( Table ) Seasonality was markedly present in the national historical series, as well as in the Southeast.

Accidental falls presented 4.5% lethality; their rates approached national ones with greater variability in the North and a predominantly upward trend in all regions. Differently than other indicators, seasonality is rare in lethality rates ( Figure 3 and Table ).

## DISCUSSION

Between 1998 and 2015, hospitalization, mortality, and lethality rates increased in Brazil. Mortality presented a more seasonal rhythm whereas hospitalizations presented a more discrete one, and rates varied among regions. The country presented an upward trend of hospitalization rate due to accidental falls for all age groups. Considering the older population (over 60 years old), the rate increased 11% for the same period. Some recent – national and international – time series studies corroborate such upward trend ^6 , 7 , 12 , 13 , 17^ . We found a higher trend of hospitalization rates due to falls among older adults than those found in the United States – a maximum of 2.3% per year – and in Australia and Brazil – both of which reported 2.7 hospitalizations/100,000 inhabitants/year ^6 , 12 , 13 , 17^ . A recent study reported an exuberant growth in the hospitalization rate due to falls among older adults in Brazil: in six years, it went from 2.58 to 41.37 per 10,000 inhabitants. It did not, however, indicate the annual growth rate ^7^ .

We found a higher growth in hospitalization rates due to falls in the Northeast region (44%), followed by the South (14%) and the Midwest (13%). It remained stationary in the Southeast (3%) and decreased in the North (48%). Another national study confirms the growth in the hospitalization coefficient for falls found in this study for all Brazilian regions, except the North ^13^ .

This upward trend in Brazilian hospitalization rates may be related to several factors, such as: population growth and ageing ^18^ ; SUS’s user awareness in expanding the active search for hospital services, when necessary; expansion and adequacy of Mobile First-Aid Services (SAMU) ^7 , 18^ ; and improvement and completeness of the IHS registry, resulting in data consolidation within SUS information systems to be more reliable. Discriminating whether the increase in hospitalization rates results from epidemiological changes or greater access of older adults to SUS hospitals is a challenge. However, we found no increase in hospitalizations for all causes among this age group in Brazil and regions, which suggests the growth of such rate for severe and moderate falls.

Victims of such accidental falls require hospital assistance or surgical treatment, occupying every time more beds ^11^ . In 2015, 41% of the hospitalizations owing to external causes were due to accidental falls in Brazil. ^13^ In Australia, they were responsible for 3% of older adult’s admission ^12^ , and, in the United States, they increased emergency visits by 27% ^17^ .

Besides incurring sequelae, falls can also cause secondary damage, such as: fear of falling, functionality decrease, loss of self-confidence, and recurrence of falls – refueling a vicious cycle. ^5^ Falls from one’s own height often result in fractures of the extremities and pelvis, followed by head and neck. But falls from greater heights, aside from the lesions mentioned, often impact the face, lung, or thorax and abdomen ^19^ .

In Brazil, 1.8% of falls result in femoral and hip fractures, 31.8% of which require surgical treatment ^9^ . These surgeries often affect individual’s independence: 40% of older adults hospitalized for falls will need caregiving help, and 10% will need help for daily activities ^5^ , and rehabilitation. Unfortunately, the severity of the fracture increases with age, and is related to a greater frailty in older adults ^19^ – the more fragile, the more likely they are to fall and suffer injuries ^17 , 20^ .

Thus, population ageing foresees an increasing morbidity and mortality from falls. Public health should emphasize falls prevention programs considering their cost-effectiveness ^21^ .

Our results suggest the need to improve, and even expand, existing falls prevention programs to specific age groups in SUS primary healthcare, mainly by using the matrix support potential of the Family Health Unit. According to worldwide guidelines ^1 , 22 , 23^ , such programs should embrace older population multidimensional assessment and intervention, including improving physical capacity by performing physical exercises that train strength, balance, and gait, by providing integrative practices, such as *tai chi chuan* ^22^ , and also by physiotherapy ^22 , 23^ . The guidelines ^22 , 23^ also recommend reducing medications, tracking falls occurrence within the population, and organizing task forces for cataract surgery ^22^ . However, increased hospitalizations is not necessarily a bad thing; it may positively represent access by improving health, preventing disease, reducing mortality, and increasing survival rates.

We also observed an increased mortality rate. At national level, it increased 32%. The highest national growth occurred among the long-lived group, with a rate of 0.67 deaths/100,000 inhabitants/month in the study period. In the Northeast and South regions, mortality rate increased in the same proportion as age. The North and Midwest inquisitively presented decreasing or stationary rates.

Such upward trend in mortality has been likewise reported in different studies conducted in Brazil, Spain, Canada, and the United States ^7 , 21 , 24 , 25^ . The first studied, conducted in Brazil, showed a 32% upward trend of mortality due to falls among older adults – similar to ours. The other studies reported lower trends, ranging between 2.5 and 15%. In the United States, mortality due to falls among older adults increased 31% in nine years of study (3% per year); in Brazil it increased 200% in six years (1996 to 2012) ^7 , 26^ . In Australia, fall-related injuries are the leading causes of death and hospitalization for accidents among older adults ^5^ .

Several factors may explain the upward trend in mortality due to falls among older adults ^21^: 1) increased incidence of falls, especially moderate and severe; 2) increased prevalence of senile frailty, underlining the association between age and mortality due to femoral fractures according to prefracture comorbidities; 3) improved quality records on deaths due to falls, although varying among regions, indicating that, in Brazil, best quality is found in the South and Southeast ^7^ .

The increase in mortality due to falls is problematic ^21^ and well discussed, considering demographic inversion and population ageing. It evinces that ageing alters individual’s functional capacities, increasing his frailty, the likelihood of falls occurrence, and reducing his recovery ability ^20 , 24^ .

In this context, integrality of healthcare plays a foundational role in the SUS. Both pre-hospital care and emergency services network must be strengthened, as they are important entries to the system for users who have suffered severe or moderate accidental fall. Hospital assistance – with physiotherapy, orthopedics, neurology, and geriatric services – should be optimized to meet the growing demand of victims of falls. Such optimization would minimize the length of stay and stimulate early mobilization and functional independence by safely using walking aids in trainings ^23^ , which will certainly reduce complications and deaths. Specialized rehabilitation centers should also be prepared to meet the demand for physiotherapy, occupational therapy, and other services for victims of accidental falls. That is, health care for people with disabilities is fundamental for integrality in the assistance of victims of falls.

Lethality indicates the severity of a certain disease and is often explored in infectious diseases and studies related to cancer. To date, we have found no studies on falls that directly analyzed this indicator. However, a study conducted by James et al ^19^ . on individuals of all ages treated in the emergency room of a New York hospital allowed us to estimate the lethality at 3.6% ^19^ – slightly lower than the one found by our study (4.44%), which included only older population.

By observing hospitalization, mortality, and lethality rates, we found seasonality in both hospitalization and mortality, with increases in the second and third trimesters, which correspond to the autumn season, March to June, and winter, June to September, in Brazil. Brazil, with its continental dimensions, hinders the generalization of weather conditions in the seasons, which varies among its regions as the country is cut by the tropic of Capricorn and the Equator along its more than 8,500,000 km ^2^ . Also, the World Health Organization (WHO) created the Falls Prevention Awareness Day, on June 24, a relevant event in Brazil for coinciding with the period of greater occurrence of falls.

We found seasonality for all age groups at national level, as well as in the Northeast, Midwest, and Southeast regions, and in the South for hospitalization rates. Such seasonality was detected both by visual analysis and by observing hospitalization rates among most or all age groups.

Mortality rates showed seasonality at national level, and for all age groups in the Southeast region. We found seasonality for the overall older population in the Northeast and Midwest, and for older adults aged 80 years or older in the Northeast. In the North, seasonality only occurred for the 70-79 years group.

Some studies associated the increase in fall-related injury rates with winter harsh weather conditions ^20 , 27 , 28^ . In Pennsylvania, the largest demand for emergency services – mainly for falls, accounting for 8% of cases ^28^ , – occurred two days after snow or ice storm. In Canada, seasonal peaks of hip fractures ^27^ (12%) occurred mainly within four to five days after the hail and snow warnings. In Australia, the highest rates of falls among older adults occurred in late Autumn and Winter (May), and were significantly lower in the Spring ^29^ . A study conducted in the South region of Brazil ^20^ observed an increase in the seasonality of hospitalizations due to falls during the Winter (May to August), when temperatures are close to zero, causing frosts, snow, and heavy rains.

Two time series studies observed seasonal mortality rates from accidental falls: the number of increased during the Winter (November and December) deaths in Canada ^25^ . Likewise, in Cuba, seasonal mortality due to falls was associated with the increase in the number of cases ^30^ during dry and cold months (December, January, and February), and in August, when the weather is warm, humid, and rainy.

Our study may contain information bias, considering the underreporting of deaths, incompleteness of IHS, and issues in data flow and consolidation in DATASUS. This limitation extends to the fact that we also selected data from the transition period between the 9th and 10th revision of the International Classification of Diseases in DATASUS.

We suggest expanding human resources for feeding and managing these information systems, and educate teams from the North, Northeast, and Midwest regions on the importance of data completeness for improving records reliability. Maintaining and intensifying falls prevention and rehabilitation programs during Autumn and Winter is of major importance not only for optimizing their functionality, but also for reducing accidental falls and their consequent hospitalizations and deaths.

We observed an upward trend in fall-related hospitalizations, mortality, and lethality among older adults between 1998 and 2015 in Brazil, and seasonal hospitalizations and mortality, marked by the increase during the second and third trimesters. We also noted that the curve patterns and values of the Southeast approached national level – a reflection of its most numerous population.
