# Supplementary material for: Increase in fall-related hospitalization, mortality, and lethality among older adults in Brazil
Source: Rev Saude Publica. 2020 Aug 10;54:76. doi: 10.11606/s1518-8787.2020054001691 (PMC7416763; doi:10.11606/s1518-8787.2020054001691)
Supplement: Translation

# Translation: Internação hospitalar, mortalidade e letalidade crescentes por quedas em idosos no Brasil

**Authors:** Lígia Raquel Ortiz Gomes Stolt, Daniel Vieira Kolisch, Clarice Tanaka, Maria Regina Alves Cardoso, Ana Carolina Basso Schmitt

**Affiliations:** I Brasil Universidade Federal da Paraíba. Centro de Ciências da Saúde. Departamento de Fisioterapia. João Pessoa, PB, Brasil; II Brasil Universidade de São Paulo. Faculdade de Medicina. Departamento de Fisioterapia, Fonoaudiologia e Terapia Ocupacional. São Paulo, SP, Brasil; III Brasil Universidade de São Paulo. Faculdade de Saúde Pública. Departamento de Epidemiologia. São Paulo, SP, Brasil

**Keywords:** Idoso, Acidentes por Quedas, Hospitalização, Mortalidade, tendências, Estudos de Séries Temporais

## Abstract

**OBJETIVO:**

Estimar as tendências de internação, mortalidade e letalidade por quedas em idosos no Brasil e regiões.

**MÉTODOS:**

Estudo descritivo realizado a partir de dados do Sistema de Informações Hospitalares do Sistema Único de Saúde. Foram incluídos os registros de todos os idosos, a partir de 60 anos, internados por quedas acidentais entre janeiro de 1998 e novembro de 2015 em todas regiões do Brasil. Foram selecionados os códigos E885, E886, E880, E884, E888 e W01, W03, W10, W17, W18 da Classificação Internacional de Doenças (CID), 9ª e 10ª revisão respectivamente, e calculadas as taxas de internação e mortalidade por quedas por 100.000 habitantes, além da letalidade. Para cálculo de tendências, usou-se o procedimento de Prais-Winsten de autorregressão para análise de séries temporais.

**RESULTADOS:**

Foram realizadas 1.192.829 internações por quedas no período, ocorreram 54.673 desfechos fatais, e a letalidade foi de 4,5%. As taxas de internação apresentaram tendências crescentes, com sazonalidade, no Brasil e nas regiões Nordeste, Centro-Oeste e Sul (taxas de crescimento de 11%, 44%, 13% e 14%, respectivamente). No Norte, a taxa de internação foi decrescente, e no Sudeste foi estacionária (taxas de crescimento: 48% e 3%).

**CONCLUSÕES:**

Houve tendência crescente de internações, mortalidade e letalidade por quedas em idosos entre 1998 e 2015 no Brasil, com picos sazonais no segundo e terceiro trimestres. É necessário aprimorar tanto a assistência hospitalar quanto incentivar programas de prevenção de quedas em idosos, visto que estamos em plena transição demográfica.

## INTRODUÇÃO

Quedas acidentais são eventos inesperados nos quais as pessoas vão de encontro ao chão, assoalho ou a um nível inferior ^1^ . Acometem cerca de 30% dos idosos acima de 60 anos e de 40 a 50% dos idosos mais velhos (acima de 80 a 85 anos), consistindo na principal causa de lesões, fatais ou não, nesses grupos, com destaque para os longevos ^3^ . São as lesões não intencionais mais frequentes nos Estados Unidos ^6^ , sendo a primeira causa de morte acidental nesse país e a terceira no Brasil ^3 , 7^ , onde sua prevalência variou entre 28,1 e 25,1% de 2011 até a atualidade ^8 , 9^ .

O avanço da idade associado à depleção natural dos sistemas fisiológicos e da funcionalidade, ao aumento da morbidade e à institucionalização precoce tornam as quedas acidentais um problema de saúde pública ^4^ , pois elas são mais frequentes na velhice e suas sequelas podem diminuir a independência funcional do idoso e aumentar o risco de morte precoce ^4^ . Além do alto custo, as internações por quedas implicam na sobrevida, pois apenas cerca de 50% dos idosos que caíram e foram admitidos em hospitais estarão vivos após um ano ^10^ .

As vítimas de quedas graves e moderadas requerem atendimento médico ou hospitalar para tratar sequelas ^11^ , gerando muitas admissões hospitalares, associadas ou não à reabilitação prolongada. Nos Estados Unidos, em 2011, elas representaram 65% das visitas nas emergências por lesões não intencionais ^6^ . Na Austrália as quedas consistiram em 3% das internações de idosos ^12^ , e no Brasil totalizaram 41% das internações por causas externas ^13^ .

A quantidade de idosos acima de 60 anos cresce mundialmente a uma taxa acelerada de 3% ao ano, ampliando o número de pessoas em risco de quedas, e consequentemente com sequelas e fatalidades ^14^ . Em 2017, a população mundial foi estimada em 7,6 bilhões de pessoas, 13% delas idosas. Em 2050, ela será de 9,8 bilhões de pessoas, das quais 21% serão idosas, no mesmo momento que o Brasil estará entre os 31 países mais populosos do mundo, os quais, em conjunto, concentrarão 75% da população global ^14^ .

Assim, há uma preocupação com o controle e cuidado com a ocorrência de quedas moderadas e graves ^11^ , pois suas sequelas frequentemente influenciam a independência e autonomia dos idosos. Ao considerarmos a importância de conhecer a distribuição e movimentação das medidas existentes de quedas para otimizar o planejamento da rede assistencial de saúde, organizando o cuidado e mantendo estrutura e recursos humanos adequados nos serviços, este estudo objetivou estimar a tendência e sazonalidade das taxas de internação, mortalidade e letalidade por quedas em idosos no Brasil e regiões.

## MÉTODOS

Neste estudo de séries temporais, utilizaram-se informações do SIH/SUS (Sistema de Informações Hospitalares do Sistema Único de Saúde) disponíveis no Departamento de Informática do Sistema Único de Saúde (Datasus), banco de dados público, gerenciado pela Ministério da Saúde, cujo uso de informações dispensa a aprovação por comitê de ética. Foram selecionados todos os registros hospitalares de idosos a partir de 60 anos, com autorizações de internação hospitalar (AIH) pagas, relativas às internações e óbitos ocorridos de janeiro de 1998 a novembro de 2015 que traziam como diagnóstico principal um dos códigos de quedas acidentais da Classificação Internacional de Doenças (CID), 9ª e 10ª revisão, respectivamente: E885, E886, E880, E884, E888 e W01, W03, W10, W17, W18. O estudo envolveu vítimas de quedas graves e moderadas, as quais requerem atendimento de emergência (tratamento de fraturas, lesões de cabeça ou interna) ou atendimento médico (feridas, contusões, entorses, cortes e abrasões), respectivamente ^11^ .

Foram calculadas as taxas específicas de internação e de mortalidade por faixa etária e região por 100.000 habitantes, além das taxas de letalidade por quedas. A letalidade é entendida como a proporção de casos de óbito entre casos da doença, indicando a gravidade da doença na população ^15^ . Também foi calculada a taxa de internação de idosos por todas as causas no Brasil e regiões, por 100.000 habitantes, para estimar a tendência ao acesso dos idosos a hospitais do Sistema Único de Saúde (SUS). O quantitativo de internações (medido pelas AIH) por quedas acidentais graves e moderadas no período, bem como o número de óbitos decorrentes delas, foram obtidos no SIH/SUS do Datasus em: Informações de saúde (TabNet) > Epidemiológicas e Morbidade > Morbidade Hospitalar do SUS (SIH/SUS) > “Causas externas, por local de residência”, considerando-se os códigos CID selecionados. O número de habitantes idosos (60 anos e mais) foi obtido a partir dos dados do Instituto Brasileiro de Geografia e Estatística, disponíveis no SIH/SUS: “População residente” no Brasil e regiões, com a idade estratificada por faixa etária em décadas (60–69, 70–79, 80 e mais anos ou longevos), além do grupo como um todo.

As tendências foram estimadas usando-se o procedimento de Prais-Winsten para regressão linear generalizada, com avaliação da autocorrelação pelo teste de Durbin-Watson. Também foram estimadas as taxas de crescimento médio, com respectivos intervalos de confiança de 95% (IC95%) ^16^ . Utilizou-se o programa Excel para importação dos dados e o Stata 13.0 para tabulação e processamento.

## RESULTADOS

No Brasil, as taxas médias de internação e de mortalidade devido a quedas no período de 1998 a 2015 foram respectivamente: 15,04 internações/100.000 habitantes/mês e 0,67 óbitos/100.000 habitantes/mês. Entretanto, não houve aumento do acesso de idosos às internações hospitalares no SUS por todas as causas no Brasil (-0,04%; IC95% -0,11–0,02) e nem em suas regiões: Norte (-0,01%; IC95%: -0,07–0,04), Nordeste (-0,05%; IC95%: -0,13–0,02), Sudeste (-0,03%; IC95%: -0,11–0,04), Sul (-0,03%; IC95%: -0,09–0,01) e Centro-Oeste (-0,02%; IC95%: -0,06–0,01).

As Figuras 1, 2 e 3 permitem observar a magnitude das taxas, bem como a tendência e a variação sazonal das mesmas. A Tabela apresenta as taxas de crescimento médio dos indicadores estudados, possibilitando identificar o tipo de tendência (crescente, decrescente ou estacionária). A presença de sazonalidade nos grupos etários está indicada pela letra “a”.

**Tabela:** Taxa de incremento anual de mortalidade, letalidade e internação de idosos por quedas no Brasil e Regiões. 1998–2015.

		Taxa de incremento anual (IC _95%_ )		
		Internação	Mortalidade	Letalidade
Brasil	Total ^b^ /60 ou +	0,11 (0,07– 0,15) ^a^	0,32 (0,28–0,36) ^a^	0,22 (0,19– 0,24) ^a^
	60–69	0,12 (0,09– 0,15) ^a^	0,17 (0,11– 0,22) ^a^	0,05 (-0,00– 0,10)
	70–79	0,11 (0,07– 0,16) ^a^	0,27 (0,22– 0,32) ^a^	0,16 (0,13– 0,19)
	Longevos ^c^	0,08 (0,01– 0,16) ^a^	0,35 (0,30– 0,40) ^a^	0,27 (0,24– 0,31)
Norte	Total ^b^ /60 ou +	-0,48 (-0,63– -0,33)	0,19 (0,03– 0,36)	0,68 (0,52– 0,85)
	60–69	0,00 (-0,14– 0,14)	0,32 (-0,44– -0,20)	0,34 (-0,53– -0,15)
	70–79	0,02 (-0,15– 0,19)	0,12 (-0,25–0,02) ^a^	0,34 (-0,49– -0,20)
	Longevos ^c^	-1,00 (-1,27– -0,72)	0,09 (-0,09–0,28)	1,03 (0,80–1,27)
Nordeste	Total ^b^ /60 ou +	0,44 (0,36– 0,51) ^a^	0,71 (0,61–0,81) ^a^	0,27 (0,20–0,34)
	60–69	0,36 (0,29– 0,43) ^a^	0,31 (0,16–0,47)	0,05 (-0,18–0,08)
	70–79	0,43 (0,35– 0,50) ^a^	0,60 (0,47–0,73)	0,16 (0,07–0,26)
	Longevos ^c^	0,55 (0,48– 0,62) ^a^	0,93 (0,80–1,06) ^a^	0,04 (-0,07–0,14)
Centro-Oeste	Total ^b^ /60 ou +	0,13 (0,06– 0,19) ^a^	0,22 (0,12–0,33) ^a^	0,14 (0,04–0,24)
	60 a 69	0,15 (0,08– 0,21) ^a^	0,19 (-0,32– -0,06)	-0,28 (-0,40– -0,16)
	70 a 79	0,12 (0,05– 0,19) ^a^	0,12 (-0,26–0,01)	0,18 (-0,31– -0,06) ^a^
	Longevos ^c^	0,62(0,53– 0,70) ^a^	0,12 (-0,03–0,27)	0,45(-0,58– -0,32)
Sudeste	Total ^b^ /60 ou +	0,03 (-0,01– 0,08) ^a^	0,23 (0,18–0,27) ^a^	0,20 (0,17–0,23) ^a^
	60–69	0,05 (0,02– 0,09) ^a^	0,13 (0,06–0,20) ^a^	0,08 (0,02–0,14) ^a^
	70–79	0,01 (-0,03– 0,06) ^a^	0,19 (0,12–0,25) ^a^	0,18 (0,14–0,23)
	Longevos ^c^	-0,07 (-0,14– 0,00) ^a^	0,17 (0,11–0,22) ^a^	0,25 (0,21–0,29) ^a^
Sul	Total ^b^ /60 ou +	0,14 (0,07– 0,21) ^a^	0,44 (0,35–0,53)	0,31 (0,23–0,39)
	60–69	0,14 (0,08– 0,19)	0,12 (-0,00–0,24)	0,00 (-0,12–0,11)
	70–79	1,25 (1,18– 1,32) ^a^	0,31 (0,19–0,42)	0,18 (0,07–0,28)
	Longevos ^c^	0,06 (-0,03– 0,16) ^a^	0,45 (0,33–0,57)	0,40 (0,31–0,49)
Tendência:		Estacionária	Crescente	Decrescente

^a^ sazonalidade / IC95%: intervalo de confiança de 95%.

^b^ Total: total de idosos do estudo, pessoas com 60 anos ou mais.

^c^ Longevos: idosos com 80 anos ou mais

As taxas nacionais de internação, mortalidade e letalidade foram crescentes no período, na maioria das regiões, exceto para a letalidade entre 60 e 69 anos, que decresceu 5%. Destaca-se que houve sazonalidade, com picos nas taxas de internação e mortalidade, no segundo e terceiro trimestres.

Houve 1.192.829 internações por quedas nos hospitais públicos/conveniados do Brasil. A tendência da taxa de internação nacional foi crescente em todos os grupos etários. As taxas de internação na região Sudeste foram superiores às nacionais. Contudo, aqui, a maior parte das taxas de crescimento indicou tendência estacionária. No Centro-Oeste a taxa de internação apresentou valores próximos ao nacional e a tendência foi crescente ( Figura 1 e Tabela ).

**Figura 1:** Taxa de internação devido a acidentes por quedas em idosos no Brasil e Regiões. 1998–2015.

As taxas de internação do Norte, Nordeste e Sul foram menores que as do Brasil. No Norte, ela foi estacionária entre 60 e 69 e entre 70 e 79 anos, e decrescente no total dos idosos e nos longevos. No Nordeste houve tendência crescente, como no Sul, apresentando-se estacionária apenas na faixa etária dos longevos. O Brasil como um todo e quatro de suas cinco regiões apresentaram sazonalidade nas taxas de internação na maioria ou totalidade dos estratos etários ( Figura 1 e Tabela ).

Foram registrados 54.673 óbitos devido às quedas acidentais durante o estudo. A taxa de mortalidade geral aumentou em todas regiões brasileiras e no Brasil, com presença de tendência crescente em todos grupos etários nacionalmente. A região Sudeste foi a única com taxas de mortalidade maiores que as do Brasil, também com tendência crescente em todos os grupos etários ( Figura 2 e Tabela ).

**Figura 2:** Taxa de mortalidade devido a acidentes por quedas em idosos no Brasil e Regiões. 1998–2015.

Nas regiões Centro-Oeste e Sul, as taxas de mortalidade foram próximas às nacionais. No Centro-Oeste a tendência foi crescente para o total de idosos e decrescente para o grupo etário de 60 a 69 anos. No Sul, todos os grupos etários apresentaram tendências crescentes.

Nas regiões Norte e Nordeste, as taxas de mortalidade foram inferiores às do Brasil. No Norte, a tendência foi crescente para o total dos idosos, decrescente para os de 60 a 69 anos e estacionária a partir dos 70 anos. O Nordeste apresentou tendência crescente em todos os grupos etários, com sazonalidade no grupo de longevos ( Tabela ). A sazonalidade foi marcadamente presente na série histórica do Brasil, assim como do Sudeste.

As quedas acidentais apresentaram letalidade de 4,5%, e suas taxas foram próximas às nacionais, com variabilidade maior na região Norte e tendência predominante crescente em todas regiões. Contrapondo os outros indicadores, a sazonalidade na letalidade é rara ( Figura 3 e Tabela ).

**Figura 3:** Taxa de letalidade devido a acidentes por quedas em idosos no Brasil e Regiões. 1998–2015.

## DISCUSSÃO

Houve aumento das taxas de internação, mortalidade e letalidade no Brasil entre 1998 e 2015, com ritmo sazonal marcante para a mortalidade e mais discreto nas internações, além de presença de diferenças regionais. A tendência da taxa de internação por quedas acidentais no Brasil foi crescente em todos estratos etários entre 1998 e 2015, especificamente de 11% de aumento no período, considerando o grupo de idosos (maiores de 60 anos). Essa tendência crescente é corroborada por alguns estudos de séries temporais recentes, nacionais e internacionais ^6 , 7 , 12 , 13 , 17^ . A tendência da taxa de internação por quedas em idosos que encontramos foi superior às encontradas nos Estados Unidos, de no máximo 2,3% ao ano, e também as da Austrália e do Brasil, ambas de 2,7 internações por 100 mil/habitantes/ano ^6 , 12 , 13 , 17^ . Destacamos um estudo recente que constatou crescimento exuberante da taxa de internação por quedas em idosos no Brasil, que passou de 2,58 para 41,37 por 10.000 idosos em seis anos, mas que não aponta a taxa de crescimento anual ^7^ .

Identificamos um maior crescimento da taxa de internação por quedas na região Nordeste (44%), seguida pela região Sul (14%) e Centro-Oeste (13%). Ela manteve-se estacionária no Sudeste (3%) e decresceu no Norte (48%). Outro estudo nacional ratifica o crescimento do coeficiente de internação por quedas encontrado em todas as regiões brasileiras, exceto no Norte ^13^ .

Essa taxa de internação crescente no Brasil pode estar relacionada a diversos fatores, como o conhecido crescimento e envelhecimento populacional ^18^ ; a maior conscientização do usuário do SUS, com ampliação da busca ativa pelos serviços hospitalares quando necessário; a ampliação e adequação de serviços de saúde móveis (SAMU) ^7 , 18^ ; e finalmente a melhoria da qualidade e completitude do registro das AIH, tornando mais fidedigna a consolidação dos dados nos sistemas de informação do SUS. Não é fácil diferenciar se o crescimento da taxa de internação se deve às mudanças epidemiológicas ou ao maior acesso de idosos aos hospitais do SUS; entretanto, constatamos que não houve aumento das internações hospitalares de idosos por todas as causas no Brasil e regiões, apontando o crescimento específico da taxa de internação por quedas graves e moderadas.

Essas vítimas de quedas graves e moderadas requerem atendimento médico hospitalar ou cirúrgico, ocupando cada vez mais leitos ^11^ . Desse modo, no Brasil, 41% das internações por causas externas em 2015 foram por quedas acidentais ^13^ . Na Austrália elas foram responsáveis 3% das internações de idosos ^12^ e nos Estados Unidos aumentaram em 27% as visitas aos setores de emergência ^17^ .

Além de gerar sequelas, as quedas ainda podem trazer danos secundários como o medo de cair, restrição funcional, perda de autoconfiança e recorrência de quedas, alimentando um ciclo vicioso ^5^ . As quedas da própria altura geralmente causam fraturas de extremidades e pélvicas, seguidas por cabeça e pescoço. Mas quando são de maiores alturas, além das lesões citadas, frequentemente atingem face, pulmão ou tórax e abdome ^19^ .

No Brasil, as fraturas de fêmur e quadril ocorrem em 1,8% das quedas, das quais 31,8% precisam de tratamento cirúrgico ^9^ . Essas cirurgias costumam afetar a independência do indivíduo, pois 40% dos idosos internados por quedas necessitarão de cuidadores domiciliares e 10% deles, de ajuda para as atividades diárias ^5^ , além da reabilitação. Infelizmente, a gravidade da fratura aumenta com a idade e se associa à maior fragilidade dos idosos ^19^ , pois, quanto mais frágil, mais susceptível a quedas e lesões ^17 , 20^ .

Dessa maneira, o envelhecimento populacional traz uma expectativa de aumento da morbimortalidade por quedas. Assim, os programas de prevenção de quedas na saúde pública devem ser enfatizados por seu custo-efetividade ^21^ .

Nossos resultados sugerem a necessidade de aprimorar e até mesmo ampliar os programas existentes de prevenção de quedas na atenção básica do SUS para grupos etários específicos, principalmente com o uso da potencialidade do apoio matricial do Núcleo de Apoio à Saúde da Família. Esses programas, conforme diretrizes mundialmente conhecidas ^1 , 22 , 23^ , devem abranger a avaliação e intervenção multidimensional do idoso, incluindo melhora da capacidade física com a realização de exercícios que envolvam treino de força, equilíbrio e marcha, mediante disponibilização de práticas integrativas como tai chi chuan ^22^ e também pelo serviço de fisioterapia ^22 , 23^ . As diretrizes ^22 , 23^ também orientam a redução do número de medicações utilizadas, rastreamento da ocorrência de quedas na população, organização de mutirões de cirurgias de catarata ^22^ , entre outros. Por outro lado, destacamos que o aumento das internações hospitalares nem sempre é ruim, pois pode ser um sinal positivo para o acesso, ao possibilitar melhora da saúde, prevenção de doenças, redução de mortalidade e aumento das taxas de sobrevivência.

A taxa de mortalidade também foi crescente em nosso estudo, apresentando aumento nacional de 32%. O maior crescimento nacional ocorreu nos longevos, com uma taxa de 0,67 óbitos/100.000 habitantes/mês no período. O aumento da taxa de mortalidade acompanhou o avanço da idade no Nordeste e Sul do Brasil. Curiosamente, no Norte e Centro-Oeste houve presença de taxas decrescentes ou estacionárias.

Essa tendência da mortalidade crescente tem sido relatada em estudos de diversos países, como Brasil, Espanha, Canadá e Estados Unidos ^7 , 21 , 24 , 25^ . No primeiro, nacional, houve uma tendência crescente de óbitos de idosos por quedas de 32%, similar à nossa, e nos demais essas tendências foram inferiores, variando entre 2,5 e 15%. Pode-se dizer também que os óbitos de idosos devido a quedas aumentaram 31% em nove anos de estudo (3% ao ano) nos Estados Unidos, e no Brasil aumentaram 200% em apenas seis anos (1996 a 2012) ^7 , 26^ . Na Austrália, as lesões por quedas consistem nas principais causadoras de morte e de hospitalização por acidentes em idosos ^5^ .

A tendência da mortalidade crescente por quedas nos idosos pode ser explicada ^21^ pela existência de diversos fatores: 1) aumento da incidência de quedas, em especial das moderadas e graves; 2) aumento da prevalência de fragilidade senil, evidenciando associação entre idade e mortalidade por fraturas de fêmur conforme comorbidades; 3) melhor qualidade do registro de mortes por quedas, ainda que exista uma variação regional, apontando que no Brasil os melhores são provenientes do Sul e Sudeste ^7^ .

Esse aumento da mortalidade decorrente de quedas é problemático ^21^ e bem discutido no que concerne à inversão demográfica e envelhecimento populacional mundial. Evidencia, portanto, que o envelhecimento altera as capacidades funcionais do indivíduo, deixando-o mais frágil, propenso à ocorrência de quedas e com capacidade de recuperação diminuída ^20 , 24^ .

Diante desse contexto, destaca-se a importância da integralidade na assistência como um dos pilares do SUS, pois é necessário fortalecer tanto o atendimento pré-hospitalar quanto a rede de urgência e emergência, visto que consistem em portas importantes de entrada no sistema para o usuário que sofreu queda acidental grave ou moderada. A assistência hospitalar, com os serviços de fisioterapia, ortopedia, neurologia e geriatria, deve ser otimizada para suprir a crescente demanda de idosos vítimas de quedas, minimizando o tempo de permanência e estimulando a deambulação e independência funcional precoce com treino de uso seguro de auxiliares para marcha ^23^ , o que certamente diminuirá as complicações e número de óbitos. Paralelamente, os centros especializados de reabilitação devem estar preparados para absorver a demanda de idosos vítimas de quedas acidentais que necessitem de fisioterapia, terapia ocupacional e outros serviços, ou seja, a rede de cuidado à pessoa com deficiência é essencial para a integralidade na assistência para as vítimas de quedas.

A letalidade denota a gravidade da doença e é usualmente explorada nas doenças infectocontagiosas e nos estudos relacionados ao câncer. Até o momento não encontramos estudos de quedas que tenham analisado diretamente esse indicador. Entretanto, a partir dos dados do estudo de James et al. ^19^ , conseguimos estimar a letalidade para indivíduos de todas as idades que foram atendidos na emergência de um hospital de Nova York em 3,6% ^19^ , ligeiramente inferior àquela do nosso estudo (4,44%), que incluiu somente idosos.

Ao observar as curvas de internação, mortalidade e letalidade, destaca-se que houve sazonalidade na internação e mortalidade, com aumentos no segundo e terceiro trimestres, ou seja, nos períodos do ano correspondentes às estações de outono (março a junho) e inverno (junho a setembro) no Brasil. Entretanto, as dimensões continentais do Brasil impossibilitam generalizar o clima nas estações do ano, pois as estações divergem de acordo com as regiões, visto que o país é cortado pelo trópico de Capricórnio e pela linha do Equador ao longo dos seus mais de 8.500.000 km ^2^ . Mesmo assim, pode-se considerar que o Dia Mundial de Prevenção de Quedas, 24 de junho, criado pela Organização Mundial da Saúde (OMS), representa um evento relevante no Brasil, pois é coincidente com o período de maior ocorrência de quedas.

Houve sazonalidade nacional, e também nas regiões Nordeste, Centro-Oeste e Sudeste em todas as faixas etárias e no Sul para a taxa de internação. Essa sazonalidade foi detectada tanto pela análise visual como pela observação da maioria ou totalidade dos estratos etários das taxas de internação.

Para a taxa de mortalidade, houve sazonalidade nacional e também em todas faixas etárias na região Sudeste. Na região Nordeste e Centro-Oeste, a sazonalidade aconteceu no grupo de idosos como um todo e também no grupo de idosos com 80 anos ou mais no Nordeste. Na região Norte ela só aconteceu para o grupo de 70 a 79 anos.

Alguns estudos têm relacionado o aumento das taxas de lesão por quedas com condições climáticas rigorosas do inverno ^20 , 27 , 28^ . A maior procura aos serviços de emergência, principalmente por quedas ^28^ , ocorreu dois dias após neve ou tempestade de gelo na Pennsylvania, representando 8% dos casos. Já os picos sazonais de ocorrência de fraturas de quadril no Canadá ^27^ (12%) ocorreram principalmente de quatro a cinco dias após os alertas de chuva de granizo e neve. Na Austrália, as maiores taxas de quedas em idosos ocorreram no final do outono e inverno (maio) e foram significativamente mais baixas na primavera ^29^ . Já no Brasil, um estudo realizado na região Sul ^20^ constatou aumento da sazonalidade das internações por quedas no inverno (maio a agosto), quando há temperaturas próximas a zero, geadas, neve e chuvas intensas.

A sazonalidade da taxa de mortalidade por quedas acidentais também foi observada em duas séries temporais. No Canadá ^25^ , verificaram número aumentado de óbitos durante o inverno (novembro e dezembro). Em Cuba, a sazonalidade da mortalidade por quedas também foi marcada pelo aumento do número de casos ^30^ , nos meses de clima seco e frio (dezembro, janeiro e fevereiro) e também em agosto, estação chuvosa, com clima úmido e quente.

É possível que haja viés de informação no estudo, por subnotificação de óbitos, incompletude de AIH e problemas no fluxo dos dados e sua consolidação no Datasus. Isso se estende ao fato de termos utilizado dados relativos ao período de tempo de transição entre a utilização da 9ª para a 10ª revisão da Classificação Internacional das Doenças no Datasus.

Recomendamos a ampliação dos recursos humanos para alimentação e gerenciamento desses sistemas de informações, sensibilizando as equipes das regiões Norte, Nordeste e Centro-Oeste para a importância da completitude dos dados para melhorar a fidedignidade dos registros. É essencial manter programas de prevenção de quedas e de reabilitação contínuos e intensificados durante o outono e inverno, visando otimizar a funcionalidade para diminuir as quedas acidentais e suas consequentes internações e óbitos.

Portanto, constatamos tendência de aumento das internações, da mortalidade e letalidade de idosos por quedas entre 1998 e 2015 no Brasil, com sazonalidade marcada pelo aumento das internações e mortalidade por quedas em idosos no segundo e terceiro trimestres. Também registramos que o Sudeste apresentou padrões de curva e valores próximos ao nacional, reflexo da sua população mais numerosa.
